# ESCRT: A Cellular Nexus of Plant Immunity and Pathogen Counter‐Strategies

**DOI:** 10.1111/mpp.70298

**Published:** 2026-06-18

**Authors:** Willam Kyle Sexton, Shunyuan Xiao

**Affiliations:** ^1^ Institute for Bioscience and Biotechnology Research University of Maryland College Park Rockville Maryland USA; ^2^ Department of Plant Science and Landscape Architecture University of Maryland College Park Maryland USA

## Abstract

The endosomal sorting complex required for transport (ESCRT) machinery consists of multiprotein complexes conserved across all eukaryotic lineages that function in several fundamental cellular processes. Among these are sorting endocytosed membrane proteins, multivesicular body (MVB) biogenesis, and autophagy. Despite the fact that these processes are also critical for plant immune responses, relatively little work has been done studying ESCRT in the context of plant immunity. Several studies indicate that ESCRT influences PAMP‐triggered immunity (PTI) and effector‐triggered immunity (ETI) by controlling the endosomal sorting of membrane‐localized or associated immune signalling components, thereby affecting their localization and stability. Very few studies have examined ESCRT's role in MVB biogenesis during pathogen infection, but because ESCRT drives the formation of MVBs, it is likely involved in all the defence responses MVBs contribute to, such as extracellular vesicle (EV) release and cell wall reinforcement. Similarly, ESCRT's involvement in autophagy during pathogen infection is understudied, but because ESCRT is responsible for phagophore sealing, it is probably required for autophagy's roles in plant immunity, which include its complicated relationship with programmed cell death. ESCRT's relevance to plant immunity is also supported by instances of pathogen effectors targeting ESCRT complexes and evidence suggesting certain ESCRT subunits are monitored by the plant immune system. In this review, we highlight evidence supporting ESCRT's importance in plant immunity, discuss the mechanisms by which ESCRT contributes to immune responses, and identify the major knowledge gaps in this area.

## Graphical abstract

The endosomal sorting complex required for transport (ESCRT) plays multifaceted roles in plants' responses to pathogens, including regulation of the localization and fate of immune receptors and other related cellular processes.

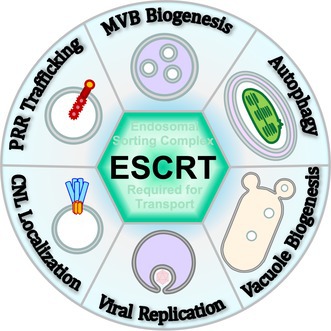

## 1 Introduction

Plants possess a sophisticated innate immune system composed of two mechanistically interconnected defence layers. The first relies on the recognition of pathogen‐associated molecular patterns (PAMPs) by cell‐surface pattern‐recognition receptors (PRRs), leading to PAMP‐triggered immunity (PTI). The second involves intracellular sensor nucleotide‐binding leucine‐rich repeat receptors (NLRs), which detect pathogen effectors delivered into host cells and activate effector‐triggered immunity (ETI) (Chisholm et al. 2006; Jones and Dangl 2006).

Most PRRs are plasma membrane (PM)‐localized receptor kinases or receptor‐like proteins with extracellular leucine‐rich repeat (LRR) or lysin motif (LysM) domains. Sensor NLRs function in the cytoplasm and are classified as coiled‐coil (CNLs) or Toll/interleukin‐1 receptor‐like (TNLs) based on their N‐terminal domains. Upon activation, some CNLs have been reported to be recruited to the PM (Bi et al. 2021; Saile et al. 2021) and/or the plant–pathogen interfacial membrane (Duggan et al. 2021). TNL‐mediated ETI also requires lipase‐like proteins (EDS1, PAD4, SAG101) and helper CNLs (ADR1s and NRG1s) with RPW8‐like coiled‐coil domains that operate downstream of TNLs. Other notable helper CNLs include the NLR required for cell death (NRC) clade in Solanaceae. PTI provides broad‐spectrum resistance, whereas ETI is typically pathogen race‐specific and often accompanied by localized programmed cell death, known as the hypersensitive response (HR) (Chisholm et al. 2006; Jones and Dangl 2006).

Together, PTI and ETI form an integrated, spatiotemporally regulated immune activation and signalling network (Ngou et al. 2022) that is increasingly recognized to depend on endomembrane trafficking mediated by cellular machineries such as the endosomal sorting complex required for transport (ESCRT) (Gu et al. 2017).

**FIGURE 1:**
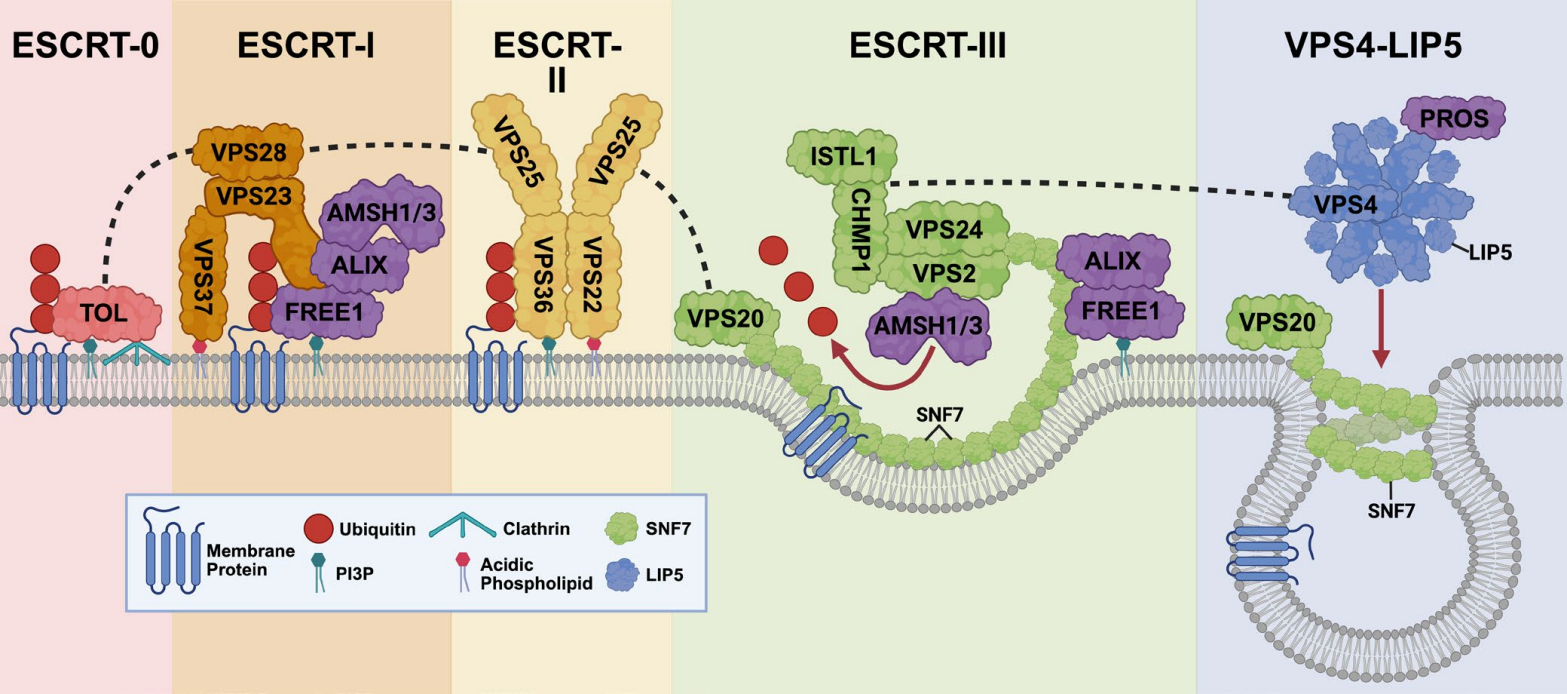
Plant ESCRT complexes. Depicted above are the plant ESCRT complexes during transport of ubiquitinated membrane proteins to intraluminal vesicles (ILVs) inside multivesicular bodies (MVBs). ESCRT‐0 binds ubiquitinated cargo proteins on early endosomes, helps cluster them, and recruits ESCRT‐I. In plants, ESCRT‐0 functions appear to be performed by TOL1‐9. ESCRT‐I binds ubiquitinated cargo and recruits ESCRT‐II to early endosomes. ESCRT‐II also binds ubiquitinated cargo and recruits ESCRT‐III. ESCRT‐III subunits form spiralled filaments on the membrane and recruit VPS4 and LIP5. VPS4 and LIP5 facilitate membrane deformation by ESCRT‐III, which drives ILV formation and ultimately ESCRT‐III dissociation from the membrane. Cargo proteins are deubiquitinated before being packaged into ILVs. PI3P: Phosphatidylinositol 3‐phosphate. Dashed lines indicate physical association between components of neighbouring ESCRT complexes.

The ESCRT machinery is a set of highly conserved multiprotein complexes that catalyse topologically membrane bending and scission events away from the cytoplasm in eukaryotes (Leung et al. 2008). Thus, ESCRT participates in a variety of cellular processes, with its best characterized role being trafficking of endocytosed PM proteins (Raiborg and Stenmark 2009). Ubiquitination of PM‐localized proteins triggers their endocytosis, after which they can be targeted for degradation in the vacuole or recycled back to the PM. The ESCRT machinery regulates this process by recognizing and clustering ubiquitinated cargo on endosomes and subsequently driving the formation of intraluminal vesicles on late endosomes that contain this cargo (Cui et al. 2016; Raiborg et al. 2002). As illustrated in Figure 1, this machinery is composed of five complexes (from ESCRT‐0 to ESCRT‐III and the VPS4‐LIP5 complex) that operate sequentially (Scheuring et al. 2011). In metazoans and fungi, ESCRT‐0 (which is composed of VPS27 and Hse1 in yeast) is the earliest acting complex and binds and clusters ubiquitinated membrane cargo before recruiting ESCRT‐I (Leung et al. 2008; Raiborg et al. 2002). ESCRT‐0 orthologs are absent in plants, but TOL (TOM1‐like) proteins (TOL1 to TOL9) appear to perform an analogous function (Korbei et al. 2013). ESCRT‐I (VPS23A/B, VPS28‐1/2, and VPS37‐1/2) and ESCRT‐II (VPS22, VPS25, VPS36) sequester ubiquitinated cargo and serve as assembly factors for ESCRT‐III (VPS20‐1/2, SNF7‐1/2, VPS24‐1/2, VPS2‐1/2/3, CHMP1A/B, VPS60‐1/2, ISTL1, CHMP7), which performs membrane deformation and scission. ESCRT‐III participates in most ESCRT‐mediated processes, particularly those involving membrane remodelling. VPS4 and its positive regulator LIP5 help drive ESCRT‐mediated membrane deformation and scission and facilitate its dissociation from membrane surfaces. Additional ESCRT components in plants include ALIX, Bro1L1, Bro1L2, AMSH1/2/3, FREE1, and PROS, with the last two thought to be unique to plants (Gao et al. 2014; Reyes et al. 2014).

ESCRT's membrane deforming and scission capabilities are also used in membrane remodelling like the sealing of autophagosomes and the formation of MVBs that are directed to the PM for secretion of exosomes (van Niel et al. 2018; Zhen et al. 2020). There is also evidence linking ESCRT to other cellular processes, such as cytokinesis, membrane repair, and vacuole biogenesis (Jimenez et al. 2014; Shahriari et al. 2010; Spitzer et al. 2006). These functions of ESCRT have been discussed extensively in other review articles (Gao et al. 2017; Isono 2021; Vietri et al. 2020; Weiner et al. 2025).

However, except for a recent review briefly discussing the role of ESCRT in the formation of extracellular vesicles (EVs) and viral manipulation of ESCRT in plant cells (Rivera‐Cuevas and Carruthers 2023), no review has yet focused specifically on ESCRT's contributions to plant immunity. Thus, this has caught our attention given the relevance of several ESCRT functions to plant immunity and the growing body of studies implicating ESCRT components in pathogen resistance (Table 1). This review examines published research on the multifaceted roles of ESCRT in plant immunity, highlighting major discoveries, identifying significant knowledge gaps, and outlining key questions for future research.

**TABLE 1:** Plant ESCRT components implicated in immunity. ESCRT subunits and accessory proteins found to be involved in plant immunity are listed alongside descriptions and sources of the evidence. Examples involving viral pathogens are not included in the table, but can be found in other reviews.

ESCRT complex	Component	Evidence	References
0	TOL3 (At1g21380)	NbTOM1 is proximal to AvrPto. Silencing results in increased susceptibility to *Pseudomonas syringae* pv. *tabaci*	Conlan et al. 2018
	TOL9 (At4g32760)	NbTOL9a is required for AVRcap1b‐mediated suppression of NRC2^H480R^ and NRC3^D480V^ autoimmunity	Derevnina et al. 2021
		*ZmTOL9* is upregulated during *Fusarium graminearum* infection	Zheng et al. 2022
I	VPS23A/B (At3g12400/At5g13860)	ZmVPS23 and ZmVPS23L bind the NLR Rp1‐D21 and prevent it from triggering a hypersensitive response (HR)	Sun et al. 2023
		VPS23B interacts with the *P. syringae* effector HopZ2 in yeast two‐hybrid (Y2H) assays	Lewis et al. 2012
		*ZmVPS23L* transcription was increased during infection by *Cercospora* *zeina* and *Cochliobolus* *heterostrophus*	Christie et al. 2017; Ding et al. 2019; Yu et al. 2022
	VPS28‐1/2 (At4g21560/At4g05000)	VPS28‐2 is involved in FLS2 sorting and flg22‐induced stomatal closure	Spallek et al. 2013
		*vps28‐2* knockout plants are more susceptible to *Hyaloperonospora arabidopsidis* Waco9, and VPS28‐2 localizes near *Phytophthora infestans* and *H.arabidopsidis* haustoria	Lu et al. 2012
	VPS37‐1/2 (At3g53120/At2g36680)	VPS37‐1 is involved in FLS2 sorting and flg22‐induced stomatal closure	Spallek et al. 2013
		Virus‐induced gene silencing (VIGS) on *ZmVPS37* reduced autoactive Rp1‐D21‐induced HR	Murphree et al. 2020
		*ZmVPS37A* expression during *F. graminearum* infection was greater in resistant *Zea mays* lines	Zheng et al. 2022
		*vps37‐1* knockout plants are more susceptible to *H. arabidopsidis* Waco9	Lu et al. 2012
III	SNF7‐1/2 (At2g19830/At4g29160)	In *Brassica napus* , *SNF7.1* and *SNF7.2* were expressed more after activation of a temperature‐resilient R protein than a temperature‐sensitive one	Noel et al. 2024
	VPS24‐1/2 (At5g22950/At3g45000)	In *Triticum aestivum* , TaVPS24 has a positive role in resistance against virulent and avirulent *Puccinia striiformis* f. sp. *tritici*	Li et al. 2024
	VPS2‐1/2/3 (At2g06530/At5g44560/At1g03950)	Overexpression of VPS2.1 with a large epitope tag that inhibits its function results in symptoms that resemble autoimmunity	Katsiarimpa et al. 2013
	CHMP1A/B (At1g73030/At1g17730)	In *B. napus* , *CHMP1A* was expressed more after activation of a temperature‐resilient R protein than a temperature‐sensitive one	Noel et al. 2024
	VPS60‐1/2 (At3g10640/At5g04850)	VPS60.1 is probably regulated positively by WRKY22, a pathogen‐responsive transcription factor	Hsu et al. 2013
		In *B. napus* , VPS60.1 is part of a protein interaction network of R proteins active against *Leptosphaeria* *maculans*	Noel et al. 2024
	ISTL1 (At1g34220)	Knocking out *LIP5* and *ISTL1* at the same time appears to result in autoimmunity	Buono et al. 2016
III	CHMP7 (At3g62080)	In *B. napus* , *CHMP7* was expressed more after activation of a temperature‐resilient R protein than a temperature‐sensitive one	Noel et al. 2024
		Overexpression of *BnCHMP7* in *Arabidopsis* resulted in autoimmunity‐like symptoms	Yang et al. 2016
VPS4‐LIP5	VPS4/SKD1 (At2g27600)	In *Nicotiana* *benthamiana*, overexpression of inactive VPS4^E232Q^ reduced cell death induced by the autoactive CNL RPM1D505V presumably by preventing stabilization of RPM1^D505V^	Schultz‐Larsen et al. 2018
		Ectopic expression of inactive VPS4^E232Q^ induced cell death in BY2 cells and *N*. *benthamiana* possibly resulting from autoimmunity	Haas et al. 2007; Schultz‐Larsen et al. 2018
		In *Oryza sativa* , loss of function of LRD6‐6, a VPS4 homologue, causes symptoms resembling autoimmunity	Yin et al. 2024
	LIP5 (At4g26750)	LIP5 is a substrate of pathogen‐responsive MPK3 and MPK6. Absence of LIP5 results in increased susceptibility to *P. syringae* pv. *tomato*	Wang et al. 2014
		LIP5 interacts with the *Ralstonia* *pseudosolanacearum* effectors RipA3 and RipAO in Y2H assays	Lewis et al. 2012; González‐Fuente et al. 2020
		Knocking out *LIP5* and *ISTL1* at the same time appears to result in autoimmunity	Buono et al. 2016
Accessory	BRO1/ALIX (At1g15130)	Powdery mildews *Podosphaera* *xanthii* and *Erysiphe* *necator* have effectors that appear to mimic ALIX	Martínez‐Cruz et al. 2018
	AMSH1 (At1g48790)	Overexpression of catalytically inactive AMSH1 inhibits ubiquitination and endocytic degradation of CERK1 triggered by AvrPtoB.	Katsiarimpa et al. 2014
		AMSH1 regulates the stability of BDA1 and is partially required for autoimmunity in the *snc2‐1D* mutant	Wang et al. 2025
		Loss of AMSH1 function results in increased susceptibility to *P. syringae* pv. *tomato* and *P. syringae* pv. *tomato* *hrcC*	Wang et al. 2025
		Reduced expression of AMSH1 resulted in increased resistance to *Erysiphe* *cruciferarum* (a biotroph) and decreased resistance to *Alternaria* *brassicicola* (a necrotroph)	Katsiarimpa et al. 2013
	AMSH3 (At4g16144)	Overexpression of catalytically inactive AMSH3 inhibits ubiquitination and endocytic degradation of CERK1 triggered by AvrPtoB	Katsiarimpa et al. 2014
		Partial loss of AMSH3 function significantly alleviates autoimmunity triggered by *pen1 syp122* and *lsd1‐2* but not *acd11*	Schultz‐Larsen et al. 2018
		Partial loss of AMSH3 function results in increased susceptibility to *P. syringae* pv. *tomato* expressing the effectors AvrRpm1 and AvrRpt2 but not AvrPphB nor AvrRps4	Schultz‐Larsen et al. 2018
		Knocking out *AMSH3* appears to cause autoimmunity dependent on TNL signalling	Schultz‐Larsen et al. 2018

## 2 Regulation of Endosomal Trafficking and Abundance of Plant Immunity Proteins by ESCRT

### 2.1 Regulation of Pattern‐Recognition Receptors and PTI

**FIGURE 2:**
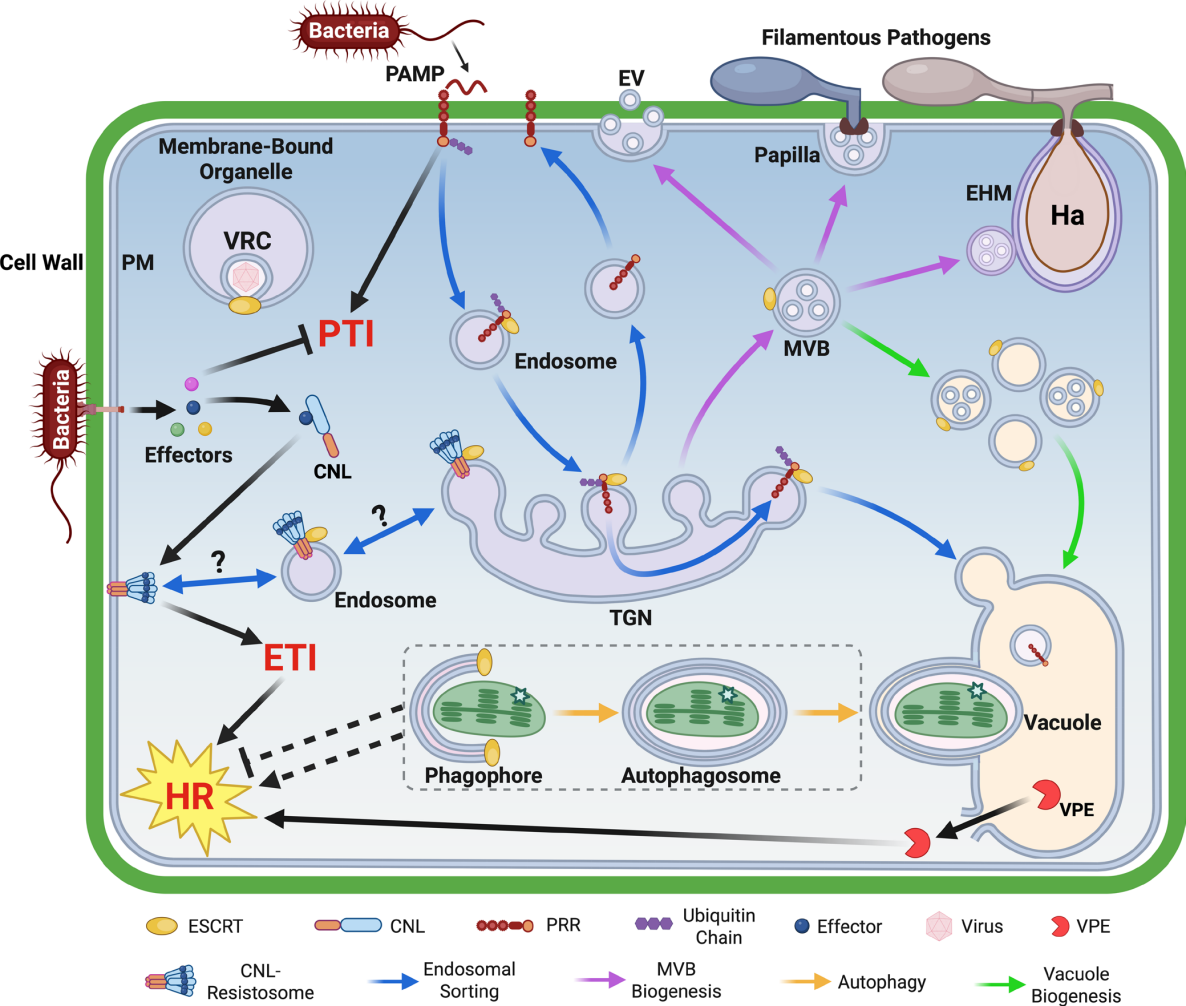
ESCRT‐mediated processes involved in plant immunity. The ESCRT machinery takes part in multiple processes related to plant immunity. Endosomal sorting (blue arrows) determines whether immunity‐related membrane proteins, like PRRs (e.g., FLS2), are recycled back to the PM or degraded in the vacuole. There is some evidence for ESCRT controlling endosomal sorting of CNLs (e.g., RPM1) as well. MVB biogenesis (purple arrows) produces MVBs that can release EVs and reinforce defence at infection sites. Autophagy (orange arrows) can play both positive and negative roles in HR. Vacuole biogenesis (green arrow) creates a large central vacuole that stores antimicrobial compounds and enzymes (e.g., VPEs) involved in HR. ESCRT also facilitates viral replication by facilitating the formation of VRCs. CNL, coiled‐coil nucleotide‐binding leucine‐rich repeat receptors; EHM, extrahaustorial membrane; ETI, effector‐triggered immunity; EV, extracellular vesicle; Ha, haustorium; HR, hypersensitive response; MVB, multivesicular body; PAMP, pathogen‐associated molecular pattern; PM, plasma membrane; PRR, pattern recognition receptor; PTI, PAMP‐triggered immunity; TGN, trans‐Golgi network; VPE, vacuolar processing enzyme; VRC, viral replication compartment.

As its name suggests, a central function of the ESCRT machinery is the sorting of proteins at endosomes, thereby regulating their cellular fate (Figure 2). Because endosomes primarily originate from the PM through endocytosis, and many PM‐resident or PM‐associated proteins such as PRRs and their co‐receptors play key roles in plant immunity, ESCRT is intrinsically positioned as a regulator of immune response. Well‐studied PRRs include FLS2, which perceives the flagellin‐derived peptide flg22, and CERK1, which recognizes chitin and chitosan, both of which activate PTI. The abundance and subcellular localization of PRRs strongly influence the immune signalling output; accordingly, PRR polyubiquitination followed by endocytosis and either recycling back to the PM or delivery to the vacuole for degradation constitute a key regulatory mechanism in plant immunity.

Spallek and colleagues presented the first piece of genetic evidence to demonstrate that ESCRT‐I components VPS28‐2 and VPS37‐1 are required for normal FLS2 sorting (Spallek et al. 2013). Following flg22 treatment, FLS2 in the *vps37‐1* mutant was endocytosed less frequently than in wild‐type plants and was localized more often to the outer membrane of MVBs. Consistent with these trafficking defects, *vps37‐1* and *vps28‐2* plants showed impaired flg22‐induced stomatal closure and increased susceptibility to *Pseudomonas syringae* pv. *tomato* (Pst) DC3000, while other flg22‐triggered defence responses, such as reactive oxygen species (ROS) production and MAP kinase activation, remained unaffected (Spallek et al. 2013). Based on these observations, the authors inferred that ESCRT‐I‐mediated sorting of FLS2 on late endosomes is specifically required for a subset of immune responses (Spallek et al. 2013).

Another study found that *vps28‐2* and *vps37‐1* mutants are also more susceptible to the oomycete pathogen *Hyaloperonospora arabidopsidis* Waco9 (Lu et al. 2012). Additionally, fluorescently labelled VPS28‐2 was observed in the vicinity of Waco9 haustoria, and both fluorescently labelled VPS28‐2 and VPS37‐1 localized around haustoria of *Phytophthora infestans* , another oomycete pathogen (Lu et al. 2012). The localization of VPS28‐2 and VPS37‐1 around the haustoria of these pathogens was similar to that of trans‐Golgi network (TGN) and early endosome markers (Lu et al. 2012). These findings highlight the importance of endosomal trafficking for plant immunity and further suggest a potential role of ESCRT in regulating the composition of membranes surrounding haustoria (Lu et al. 2012).

Among the most important ESCRT‐related proteins are deubiquitinases (DUBs), a class of enzymes that remove ubiquitin from substrate proteins. ESCRT‐associated DUBs can act at early ESCRT complexes to remove ubiquitin from cargo proteins, facilitating recycling back to the PM. They also function at ESCRT‐III to recycle ubiquitin from cargo proteins before they are packaged into MVBs destined for degradation in the vacuole (Clague et al. 2012; Agromayor and Martin‐Serrano 2006). These DUBs may also regulate ESCRT function by deubiquitinating ESCRT subunits (Row et al. 2006; Sierra et al. 2010). In plants, the ESCRT‐associated DUBs are homologues of AMSH (associated molecular with SH3 domain of STAM), one of the major ESCRT‐associated DUBs in mammals (Agromayor and Martin‐Serrano 2006).

Interestingly, it was reported that overexpression of catalytically inactive AMSH1 or AMSH3 in *Arabidopsis* alleviates AvrPtoB‐dependent degradation of CERK1 (Katsiarimpa et al. 2014). Given that AvrPtoB is a bacterial effector that mimics a plant E3 ubiquitin ligase (Rosebrock et al. 2007), this finding suggests that AMSH1 and AMSH3 are required for ubiquitin‐mediated degradation of PRRs (Katsiarimpa et al. 2014). At first glance, this result may seem paradoxical as overexpressing catalytically inactive forms of AMSH1 and AMSH3 should inhibit endogenous DUB activity, potentially leading to increased ubiquitination and enhanced degradation of CERK1. A plausible explanation is that functional impairment of AMSH1 and AMSH3 may disrupt ESCRT machinery activity, thereby inhibiting trafficking of the ubiquitinated CERK1 to the vacuole for degradation (Katsiarimpa et al. 2014). This would be consistent with other studies showing that expression of dominant‐negative ESCRT subunit mutants interferes with membrane protein degradation (Cai et al. 2014; Katsiarimpa et al. 2013). Accordingly, these findings provide evidence for a functional connection between ESCRT‐associated DUBs, ESCRT activity, and regulation of vacuolar trafficking in plant immunity.

A recent report also implicated AMSH1 in PTI, probably through positive regulation of the stability of Bian Da 1 (BDA1) in *Arabidopsis* (Wang et al. 2025). BDA1 is an ankyrin‐repeat transmembrane protein required for autoimmunity triggered by a gain‐of‐function mutation in a receptor‐like protein SNC2 (suppressor of npr1‐1, constitutive) (Yang et al. 2012). Consistently, *amsh1* single mutants showed compromised PTI and basal resistance in restricting growth of the Pst DC3000 *hrcC* mutant (which is defective in effector secretion) and the wild‐type Pst DC3000, respectively (Wang et al. 2025).

Collectively, the findings described above support the notion that PRRs and their transmembrane partners undergo endocytosis upon activation/stimulation, and that their fate following endocytosis is regulated by the ESCRT machinery (Figure 2). As mentioned previously, one possible fate for endocytosed PM proteins is to be packaged into MVBs by ESCRT and trafficked to the vacuole for degradation. It is unclear exactly how ESCRT helps direct PM proteins to subcellular locations other than the vacuole, but one conceivable way is that deubiquitination of endocytosed PM proteins by ESCRT‐associated DUBs at TGNs allows PM proteins to dissociate from the ESCRT machinery and be captured by mediators of other trafficking pathways, such as adaptor protein complex 1 (AP‐1) (Gravotta et al. 2019). Perhaps the timing of this deubiquitination helps determine which trafficking pathway PM proteins take, or maybe deubiquitination at an early enough time point simply prevents PM protein trafficking to the vacuole without directing them toward a specific alternate pathway. Another possibility worth considering is that the fate of endocytosed PM proteins is affected by which TOLs or combinations of TOLs bind to them after endocytosis.

### 2.2 Regulation of Intracellular NLRs and ETI

A study in maize by Sun et al. found that ESCRT can also regulate the trafficking of a CNL. ESCRT‐I subunit ZmVPS23 and its homologue ZmVPS23L were found to bind the coiled‐coil domain of the CNL Rp1‐D21 and sequester it in endosomes away from the nucleocytoplasm where it apparently needs to localize to induce HR (Sun et al. 2023). While ZmVPS23 and ZmVPS23L can repress HR triggered by Rp1‐D21 in both maize and *Nicotiana benthamiana*, they failed to repress HR in *N*. *benthamiana* induced by transient expression of the CNLs MLA10 (from barley) and RPM1^D505V^ (from *Arabidopsis*) (Sun et al. 2023). These results suggest that not all CNLs are regulated by ESCRT‐I in this manner, or alternatively, that this mode of regulation may be unique to maize. Interestingly, another ESCRT‐I subunit in maize, ZmVPS37, has a positive role in Rp1‐D21‐induced HR (Murphree et al. 2020). This is the same ESCRT‐I subunit that plays a positive role in flg22‐triggered PTI (Spallek et al. 2013). *ZmVPS23L* and *ZmVPS37* in maize have also been found to be upregulated during infection by various pathogens, such as *Cercospora zeina* (grey leaf blight), *Cochliobolus heterostrophus* (southern leaf blight), and *Fusarium graminearum* (Fusarium head blight), suggesting ESCRT‐I is commonly upregulated during infection (Christie et al. 2017; Ding et al. 2019; Yu et al. 2022; Zheng et al. 2022). These studies suggest that ESCRT‐I is capable of both positively and negatively regulating immune signalling, apparently by controlling the localization of signalling proteins.

The ESCRT‐0‐like component NbTOL9a has also been found to negatively regulate HR. Evidence from RNAi and overexpression experiments indicates that NbTOL9a partially suppresses HR‐like programmed cell death (PCD) triggered by autoactive helper CNLs NRC2^H480R^ and NRC3^D480V^ but not the autoactive helper CNL NRC4^D480V^, the autoactive NRC‐independent CNL NbZAR1^D481V^, nor the autoactive mitogen‐activated protein kinase kinase (MAPKK) MEK2^DD^ (Derevnina et al. 2021). These results indicate that NbTOL9a (and possibly ESCRT in general) may operate at or downstream of some CNL signalling nodes but not others. It is unclear how NbTOL9a regulates these helper CNLs, but apparently it involves very transient or no physical interaction (Derevnina et al. 2021).

Results from another study implicate ESCRT in ETI activated by CNLs and autoimmune symptoms caused by gain‐of‐function mutations in CNLs. In an elegant study, Schultz‐Larsen et al. showed that AMSH3 is required for autoimmunity, including reduced plant stature and spontaneous HR‐like cell death, triggered by the loss of the homologous syntaxins SYP121 and SYP122 in *Arabidopsis* (Schultz‐Larsen et al. 2018). Although the relevant substrate(s) of AMSH3 were not identified in this context, the same study demonstrated that AMSH3 activity is also required for ETI mediated by the CNLs RPS2 and RPM1, but not for ETI activated by RPS5 (also a CNL) or RPS4 (a TNL) (Schultz‐Larsen et al. 2018). Schultz‐Larsen et al. also provided genetic evidence that AMSH3 is required for helper CNL ADR1‐L1 and ADR1‐L2‐dependent HR‐PCD in the *lsd1‐2* mutant but not required for TNL‐dependent HR‐PCD in the *acd11* mutant (Schultz‐Larsen et al. 2018). Interestingly, the authors also found that overexpression of catalytically inactive SKD1/VPS4^E232Q^ in *N. benthamiana* reduced cell death caused by expression of the autoactive CNL RPM1^D505V^ (Schultz‐Larsen et al. 2018). Collectively, these findings strongly suggest that ESCRT plays an important positive role in CNL‐mediated ETI and the associated HR‐PCD, possibly by controlling the fate or abundance of a subset—but not all—CNL receptors (Figure 2).

The mechanism by which ESCRT regulates NLRs and ETI is unclear, but multiple possibilities are conceivable. NLRs are partly regulated by ubiquitin‐mediated degradation (Gao et al. 2022), so it is possible ESCRT helps regulate their abundance in a manner similar to PM proteins as described in the previous section. For some NLRs like the *Arabidopsis* CNL L5 (Huang et al. 2021), the 26S proteasome has been implicated in their degradation suggesting ESCRT is not involved, but in many other cases the mechanism of degradation has not been identified, leaving open the possibility of ESCRT‐mediated vacuolar degradation. Another potential mechanism is that ESCRT regulates ETI indirectly by controlling the abundance and localization of PM‐associated immune signalling proteins (like PRRs) that are required for NLR‐mediated immunity (Yuan et al. 2021). However, this explanation does not account for observed changes of NLR localization that are dependent on ESCRT (Sun et al. 2023). More research is needed to identify how ESCRT regulates NLRs' abundance and localization.

Another mystery regarding ESCRT's regulation of NLRs and ETI is why it is involved in the signalling of some CNLs but not others. Reliance on NDR1 is probably not a determining factor because RPS2, RPM1, and RPS5 all require NDR1 for signalling (Day et al. 2006), but only ETI triggered by RPS2 and RPM1 were disrupted in *amsh3* mutants (Schultz‐Larsen et al. 2018). One possible explanation could be that ESCRT regulates CNLs at some subcellular locations but not others. Sensor and helper CNLs have been found to localize to various places including the PM, chloroplasts, mitochondria, endoplasmic reticulum, and nucleus (Alam et al. 2021; Saile et al. 2021; Ibrahim et al. 2026). The ESCRT machinery appears to mainly regulate proteins at the PM and in the endomembrane system, so it may not influence CNLs that primarily localize to other places. Another possibility is that ESCRT only regulates CNLs with certain types of ubiquitin modifications. This is plausible because at least some TOLs (i.e., TOL2 and TOL6) have a preference for K63‐linked polyubiquitin (Moulinier‐Anzola et al. 2020). The type of ubiquitin modification added to a protein is determined by the ubiquitin ligases that target it, and ubiquitin ligases that target NLRs (e.g., SNIPER1, BOI) appear to have some level of specificity (Wu et al. 2020; Huang et al. 2021), so it is possible that hypothetical ubiquitin ligases that add K63‐linked polyubiquitin to NLRs only target a subset of CNLs. Testing additional CNLs for ESCRT‐dependency would be helpful in solving this mystery.

Additional work is also required to determine why ESCRT appears to be uninvolved in TNL signalling. Perhaps ESCRT is more important for CNL signalling because, unlike TNLs, activated CNLs localize to membranes where they may be targeted by ESCRT, which is membrane‐associated. Notably, several sensor CNLs (e.g., RPM1 and RPS2) (Alam et al. 2021), as well as the helper CNLs ADR1‐L1 and ADR1‐L2 (Saile et al. 2021), predominantly localize to the plasma membrane, whereas TNLs remain predominantly nucleocytoplasmic (Bernoux et al. 2023). If true, however, this would raise the question of why ESCRT does not regulate TNL signalling by controlling the localization of membrane‐associated helper CNLs that operate downstream of TNLs. The answer could be related to the subcellular localization patterns of NRG1‐type helper CNLs, which appear to differ from those of ADR1‐type helper CNLs and sensor CNLs. In contrast to canonical CNLs, which mostly localize to the PM, NRG1 helper CNLs localize to organelles like chloroplasts and mitochondria, where they may not be subject to ESCRT control (Alam et al. 2021; Saile et al. 2021; Ibrahim et al. 2026). Activated TNLs signal through both ADR1 and NRG1 modules (Sun et al. 2021), so while ESCRT dysfunction appears to compromise ADR1‐dependent signalling, it may not affect NRG1‐dependent signalling, which can still lead to the development of ETI. More studies are needed to test this hypothesis.

### 2.3 Regulation of Atypical Membrane‐Localized Disease Resistance Proteins

In addition to classical NLR‐type immune receptors, plants also deploy atypical disease resistance proteins. One example is the wall‐associated kinase‐like 10 (WAKL10) family, which confers resistance to *Leptosphaeria maculans*, the fungal pathogen responsible for blackleg disease in *Brassica* crops. In a study investigating the basis of temperature sensitivity in this resistance pathway in *Brassica napus* , Noel et al. reported that several ESCRT‐III components—SNF7.1, SNF7.2, CHMP7, and VPS46.2/CHMP1A—were more highly expressed at the mRNA level during immune responses triggered by a temperature‐resilient WAKL10 variant than by a temperature‐sensitive variant (Noel et al. 2024). In addition, the likely existence of a protein–protein interaction network including ESCRT‐III subunit VPS60.1 in the temperature‐sensitive variant led the authors to speculate that ESCRT contributes to WAKL10 trafficking (Noel et al. 2024). This network had a regulatory interaction with the pathogen‐responsive transcription factor WRKY22, which also targets the VPS60.1 promoter in *Arabidopsis* (Hsu et al. 2013), suggesting upregulation of ESCRT‐III may be a common response during pathogen infection.

## 3 ESCRT Regulation of Membrane Shaping Processes Associated With Immunity

### 3.1 MVB Formation and EV Release

The ESCRT machinery plays a central role in the generation of MVBs, including the formation of intraluminal vesicles (ILVs) which is driven by late‐acting ESCRT components (i.e., ESCRT‐III and VPS4) (Henne et al. 2013) (Figure 2). Many studies have shown that MVB biogenesis is pathogen inducible in plants and that MVBs accumulate near sites of pathogen attack (An et al. 2006; Wang et al. 2014). These infection‐associated MVBs can fuse with the PM with the help of Rab GTPases and SNAREs and release small EVs, specifically exosomes, that contain proteins involved in defence (like antifungal proteins) and ROS signalling (like phospholipases), suggesting roles in both direct antimicrobial defence and transmitting immune signals to neighbouring cells (Kowal et al. 2014; Movahed et al. 2019; Rutter and Innes 2017) (Figure 2). Notably, during attempted cell wall penetration by filamentous fungal and oomycete pathogens, small EVs can fuse with one another to form paramural bodies (PMBs), which further contribute to the formation of pathogen‐induced, callose‐rich papillae at the site of penetration to block the invasion (An et al. 2006; Nielsen et al. 2012) (Figure 2). For additional information about this, we direct readers to a recent review on the role of endomembrane trafficking in defence against powdery mildews (Thordal‐Christensen et al. 2026). Plant EVs have also been demonstrated to deliver small RNAs to attacking pathogens to silence pathogenicity genes through RNA interference (RNAi) (Cai et al. 2018). Interestingly, the plant‐specific ESCRT component FREE1 has been shown to negatively regulate miRNA biogenesis by disrupting the microprocessor complex in the nucleus, so ESCRT appears to also regulate RNAi in a way independent of EV release (Li et al. 2023). On the other hand, host plant EVs can also benefit the cell–cell movement of certain viruses (see section 4.1). For example, turnip mosaic virus (TuMV) appears to use them to spread inside infected plants (Movahed et al. 2019). This and the many other roles of EVs in plant immunity have been reviewed extensively in other articles (e.g., Liu et al. 2021; Zhou et al. 2022).

While ESCRT's function in generating MVBs directed to lysosomes or vacuoles for cargo degradation is well established, its role in generating MVBs and EVs involved in pathogen defence remains less well studied. One study found that PTI signalling is directly connected to ESCRT through LIP5, which is a late‐acting ESCRT component. LIP5 is phosphorylated by the pathogen‐responsive MPK3 and MPK6 and enhances the ATPase activity of VPS4, which disassembles ESCRT‐III from membranes, the final step in MVB formation/maturation (Wang et al. 2014). Consistent with this role, loss of LIP5 results in reduced formation of MVBs and EVs following Pst DC3000 infection and increased growth of Pst DC3000, suggesting that EVs originating from ESCRT‐produced MVBs play an important role in defence against bacterial pathogens (Wang et al. 2014). However, PR1 secretion after Pst DC3000 infection remained unaffected in the *lip5‐1* mutant, suggesting that LIP5 and MVB formation are not required for all defence‐related secretion (Wang et al. 2014). This is unsurprising because the conventional secretory pathway does not require ESCRT and there may also be ESCRT‐independent pathways for MVB formation (Theos et al. 2006).

Collectively, late endosomes, MVBs, EVs, and PMBs probably represent spatiotemporally interconnected, membranous structures that are mobilized to counter pathogen invasion within the extracellular space between the plasma membrane and the cell wall. However, the specific steps at which ESCRT functions within this continuum, and the mechanisms by which it regulates these processes in plants, remain largely unexplored.

### 3.2 Autophagy

Autophagy is a highly conserved cellular housekeeping process that mediates bulk degradation of cytoplasmic material, eliminating damaged or dysfunctional cellular components, such as organelles, while recycling important nutrients. Although autophagy is known to participate in responses to a wide variety of stresses including pathogen challenge, the reported roles of autophagy in plant immunity are complex and sometimes contradictory (Sertsuvalkul et al. 2022). This complexity is exemplified by evidence that autophagy contributes to both the induction of ETI‐associated HR‐PCD and its restriction to the site of infection. For example, ETI‐induced HR‐PCD is suppressed in *Arabidopsis* *atg7* and *atg9* mutants following infection with avirulent Pst strains (Hofius et al. 2009). In contrast, silencing key autophagy components such as ATG3, ATG6, or ATG7 in *N. benthamiana* results in unrestricted HR‐PCD during ETI (Liu et al. 2005). Autophagy also plays a pro‐survival role in inhibiting necrotic cell death and death associated with cell stress (Sertsuvalkul et al. 2022). A direct molecular link between autophagy and PTI was identified when BAK1 was found to phosphorylate ATG18a during infection by *Botrytis cinerea*, a necrotrophic pathogen, suppressing autophagosome formation and dampening resistance (Zhang et al. 2021). Autophagy also plays regulatory roles in selective degradation of pathogen effectors, viral proteins, and host immunity components besides immune receptors (Leong et al. 2022).

ESCRT's intrinsic role in autophagy is reflected by the recruitment of ESCRT components to the phagophore and the occurrence of autophagy defects when such ESCRT components are depleted in plant cells (Takahashi et al. 2018; Zhen et al. 2020) (Figure 2). Additional connections between ESCRT and autophagy include ESCRT's involvement in microautophagy at late endosomes and the occasional fusion of autophagosomes with late endosomes carrying membrane proteins sorted by ESCRT into the vacuolar/lysosomal degradation pathway. Interestingly, a recent study hinted that fusion of autophagosomes and late endosomes may be involved in degradation of pathogen effectors (Zhu et al. 2025). The requirement of ESCRT for autophagy means that ESCRT can be inferred to take part in all the immunity‐related processes involving autophagy. However, reports showing direct causal relationships between disruption of ESCRT and misregulation of autophagy plus alteration of plant immunity are rare, probably due to the above‐described complex relationship between autophagy and immunity. In one of the few such studies, reduced expression of the ESCRT‐associated DUB AMSH1 in *Arabidopsis* was shown to cause defects in autophagosome trafficking to the vacuole as well as increased resistance to *Erysiphe cruciferarum* (a biotrophic pathogen causing powdery mildew disease) and enhanced susceptibility to *Alternaria brassicicola* (a necrotrophic pathogen) (Katsiarimpa et al. 2013). The authors attributed these altered infection phenotypes to dysregulation of salicylic acid (SA) signalling which plays an important role in immunity and PCD and is negatively regulated by autophagy, but they also acknowledged the possibility that ESCRT may play more direct roles in plant immunity (Katsiarimpa et al. 2013; Yoshimoto et al. 2009). These results are consistent with the general finding that autophagy plays a negative role in defence against biotrophic pathogens and a positive role against necrotrophic ones as a result of its negative regulation of death associated with necrosis and cell stress (Sertsuvalkul et al. 2022). In another study, loss of function of CFS1, which interacts with ESCRT‐I subunit VPS23A and localizes to ESCRT‐I‐positive late endosomes, resulted in reduced autophagosome turnover and autoimmunity‐associated HR‐PCD (Sutipatanasomboon et al. 2017). The authors found that HR‐PCD in the *cfs1* mutant is dependent on EDS1 and likely an unknown TNL but did not determine what specifically triggers autoimmunity in this instance, whether it be disruption of autophagy, disruption of another ESCRT‐mediated process, or triggering of a TNL that guards CFS1 (Sutipatanasomboon et al. 2017). Autophagy's conflicting roles in immunity mean that the effects of its disruption may vary depending on the context (e.g., plant age, pathogen type). Thus, understanding the precise molecular connections between ESCRT, autophagy, and immune regulation requires high‐resolution, spatiotemporal dynamic studies preferably with inducible perturbation of the ESCRT and/or the autophagy machinery.

### 3.3 Vacuole Morphology and Biogenesis

In addition to serving as the site for targeted degradation of membrane proteins involved in immune activation and regulation, plant vacuoles contribute to immunity by acting as reservoirs for defence proteins, secondary metabolites, and hydrolases that are released upon pathogen attack (Madina et al. 2019; Sarwar et al. 2024). Some of the defence compounds stored in the vacuole, like cyanogenic glycosides, are inactive precursors of chemicals that are converted into toxic molecules, such as hydrocyanic acid (HCN), which are directly harmful to pathogens (Freeman and Beattie 2008). Others include enzymes, like myrosinases, that produce antimicrobial compounds such as isothiocyanates following vacuole release (Kissen et al. 2009). In addition, vacuolar processing enzymes (VPEs), a group of hydrolytic enzymes, play an important part in the initiation of PCD, including HR‐associated PCD (Sarwar et al. 2024).

ESCRT has been inferred to play a role in vacuole biogenesis, particularly through its function in endosomal maturation and MVB formation, which are essential precursors to functional vacuoles, thereby influencing vacuolar content and membrane composition (Figure 2). Genetic mutations in ESCRT components *ALIX*, *FREE1*, and *AMSH3* have been reported to cause small, fragmented vacuoles instead of a single, large, central vacuole although the precise mechanisms by which ESCRT controls vacuole biogenesis and structure is still poorly understood (Isono et al. 2010; Kalinowska et al. 2015; Kolb et al. 2015). Whether defects in vacuole morphology impair the release of defence compounds from the vacuole remains unclear; therefore, the contributions of ESCRT to immunity via its role in vacuole biogenesis are unknown but potentially worth exploring.

## 4 Pathogen Manipulation of ESCRT

### 4.1 Viral Replication Compartment (VRC) Formation

The most well‐documented role of ESCRT in plant and animal pathogenesis is facilitating the formation of viral replication compartments/complexes (VRCs) (Figure 2). VRCs are compartments derived from host membranes that serve as sites for virus replication and assembly. The majority of plant viruses are positive‐strand RNA viruses (+ssRNA) that require VRCs for their multiplication (Hyodo and Okuno 2016). Most cellular compartments can serve as sources for VRCs, including mitochondria and peroxisomes, and targeting to specific organelle membranes is apparently not a strong requirement for viruses (Laliberté and Sanfaçon 2010). Evidence indicates that plant viruses hijack multiple components of the endomembrane trafficking machinery, including SNAREs and COPII, to build VRCs; however, the strongest evidence supports a central role for ESCRT machinery that remodels host membranes to generate VRCs (Agaoua et al. 2021). For example, in both melon ( *Cucumis melo* ) and common bean ( *Phaseolus vulgaris* ), mutations in *VPS4*, a late‐acting ESCRT component, were associated with increased resistance to certain viruses; however, it remains unclear whether this is the result of altered function of VPS4 or inability of the viruses to recruit VPS4 (Agaoua et al. 2022; Soler‐Garzón et al. 2021).

Overexpression of dominant negative ESCRT components has also been shown to inhibit viral replication. For instance, overexpression of dominant‐negative *AtVPS4* ^*E232Q*^ in *N. benthamiana* disrupted replication of carnation Italian ringspot virus (CIRV) and (TBSV) but not tobacco rattle virus (TRV) (Richardson et al. 2014). Similarly, overexpression of dominant‐negative *AtSNF7* containing large epitope tags in *N*. *benthamiana* resulted in reduced reproduction of brome mosaic virus (BMV) but not (TRV) or tobacco mosaic virus (TMV) (Diaz et al. 2015).

More direct evidence for the recruitment of ESCRT by plant viruses has been reported in several studies. During CIRV infection of *Nicotiana tabacum* BY‐2 cells, VPS23A is recruited to mitochondria, the site of CIRV replication, by the viral protein p36 (Richardson et al. 2014). TBSV replication protein p33 has also been shown to interact with Vps4p, as well as various ESCRT‐III components, in yeast and recruit it to peroxisome‐derived VRCs (Barajas et al. 2014). Thus, the success of viral replication in many cases relies on the ability of the virus to co‐opt the host ESCRT machinery, indicating that they can serve as important susceptibility factors during viral infection. The role of ESCRT in viral replication in plants is discussed in greater detail in other reviews (Agaoua et al. 2021; Hyodo and Okuno 2016; Rivera‐Cuevas and Carruthers 2023).

### 4.2 Pathogen Effectors Targeting ESCRT

Compelling evidence supporting an important role of ESCRT in plant immunity comes from the discovery of pathogen effectors that target ESCRT components. Given that effectors act to suppress host immunity and facilitate pathogen proliferation, these observations strongly suggest that ESCRT is an important contributor to immune regulation. Below, we highlight several illustrative examples.

AVRcap1b, an effector from *P. infestans* , suppresses autoimmunity caused by the autoactive NLRs NRC2^H480R^ and NRC3^D480V^ in *N*. *benthamiana* in a manner dependent on the ESCRT‐0‐like subunit NbTOL9a, with which AVRcap1b physically interacts (Derevnina et al. 2021). This suggests that AVRcap1b inhibits NRC2/3 associated cell death by manipulating early‐acting ESCRT machinery (Derevnina et al. 2021). An effector from *P. syringae* pv. *tabaci*, AvrPto, was found to be proximal to another putative ESCRT‐0‐like protein in *N*. *benthamiana* with 54% amino acid homology to *Arabidopsis* TOL3 (Conlan et al. 2018). Although this protein does not physically interact with AvrPto, it associates with NbAPK1, another protein proximal to AvrPto, suggesting that it is part of a protein complex targeted by AvrPto (Conlan et al. 2018). The roles of these two genes in immunity were validated by the observation that their silencing compromised resistance to *P. syringae* pv. *tabaci* (Conlan et al. 2018). The functions of AVRcap1b and AvrPto may be to disrupt the endosomal sorting of membrane‐bound immune signalling components by manipulating ESCRT‐0 analogs.

In another example, PEC034, a candidate effector from the powdery mildew pathogen *Podosphaera xanthii*, was predicted to act as an ALIX‐like protein, leading the authors to postulate that it mimics endogenous ALIX to interfere with host cellular trafficking machinery (Martínez‐Cruz et al. 2018). The role of PEC034 as a bona fide effector was validated by host‐induced gene silencing, which resulted in a marked reduction in *P*. *xanthii* growth (Martínez‐Cruz et al. 2018). A putative effector in *Erysiphe necator*, another powdery mildew species, shares structural similarity with PEC034, suggesting that it may also target ESCRT machinery (Martínez‐Cruz et al. 2018). It has been established that ALIX helps control the protein level of the abscisic acid receptor PYL4, raising the possibility that these effectors may imitate ALIX to manipulate the levels of receptors involved in immunity (García‐León et al. 2019).

Additionally, yeast two‐hybrid assays have been used to identify putative interactions between VPS23B and the *P. syringae* type III effector HopZ2 as well as between LIP5 and RipA3 and RipAO from *Ralstonia pseudosolanacearum* (González‐Fuente et al. 2020; Lewis et al. 2012). Given that VPS23B recruits ALIX to ESCRT‐I, HopZ2 may function analogously to PEC034 from *P. xanthii*. Because LIP5 functions to facilitate the formation of MVBs, *R. pseudosolanacearum* may deploy RipA3 and RipAO to interfere with this process, compromising the release of EVs and PMBs.

Collectively, these observations demonstrate that pathogens have evolved effectors that target ESCRT machinery, probably to disrupt endosomal sorting of membrane‐localized immune signalling proteins and EV release, thereby subverting host immunity.

## 5 Disrupting ESCRT Can Cause Autoimmunity

Important components of plant immune systems are sometimes monitored, or guarded, by NLRs that trigger a strong immune response when those components are missing or altered in some way. Manipulation of immunity signalling components is generally an indication that an adapted pathogen is present and releasing effectors into the cell. Thus, autoimmunity resulting from functional impairment of a protein strongly suggests that it may be an important immunity component.

The fact that individual disruptive mutations in multiple ESCRT subunits result in autoimmunity suggests that certain components of the ESCRT machinery are guarded by NLRs because they are important for plant immunity. *AMSH3* was originally believed to be an essential gene because *amsh3* null mutations and overexpression of catalytically inactive *AMSH3* ^*AXA*^ results in developmental defects and seedling lethality (Isono et al. 2010). However, subsequent work revealed that the lethality of *amsh3* null mutations is EDS1‐dependent, implying that AMSH3 is monitored by TNLs that signal through the EDS1 node (Schultz‐Larsen et al. 2018). As mentioned in an earlier section, mutant alleles of the ESCRT‐I interactor *CFS1* cause autoimmunity that is also dependent on EDS1 and thus may also be guarded by TNLs (Sutipatanasomboon et al. 2017). In the latter case, whether CFS1 itself or an ESCRT‐I subunit is the guarded target remains unknown. A study also found that simultaneously knocking out *LIP5* and *ISTL1* results in a phenotype resembling autoimmunity, that is, stunted growth, spontaneous cell death, ROS accumulation, and elevated *PR1* expression (Buono et al. 2016). This phenotype was greatly alleviated when the plants were grown at a higher temperature (28°C instead of 22°C) (Buono et al. 2016). Elevated temperatures have been shown to dampen plant immunity and suppress autoimmunity defects by altering NLR activity and diminishing SA signalling (Li et al. 2020; van Wersch et al. 2016; Zhu et al. 2010). The dependence of the *ist1‐1 lip5‐1* mutant defects on temperature suggests they are likely the result of autoimmunity. However, it has also been proposed that ESCRT cargo could be what is actually monitored by NLRs and not the ESCRT machinery itself (Schultz‐Larsen et al. 2018). It is possible that disruption of ESCRT alters the activity, amount, or localization of a protein that is monitored by the plant immune system; thus, disrupting ESCRT may indirectly lead to autoimmunity.

Other studies have found more ambiguous evidence that disrupting ESCRT results in autoimmunity. Overexpression of VPS2.1 with a C‐terminal GFP tag, which inhibits its function, in *Arabidopsis* caused a dwarf phenotype accompanied by early senescence and increased expression of *PR1* and *SAG13* (Katsiarimpa et al. 2013). Overexpression of *CHMP7* from *B. napus* in *Arabidopsis* also resulted in early senescence and upregulation of *SAG101* (Yang et al. 2016). Ectopic expression of catalytically inactive AtVPS4/SKD1^E232Q^ resulted in cell death in *N*. *benthamiana* and *N. tabacum* BY‐2 cells (Haas et al. 2007; Schultz‐Larsen et al. 2018). A mutation in the *VPS4/SKD1* homologue of *Oryza sativa* (*Lrd6‐6*) that disrupts its ATPase activity and interactions with ESCRT components SNF7.1/7.2/7.3 caused accumulation of ROS, spontaneous cell death, and elevated *PR* gene transcription levels (Yin et al. 2024). The evidence in these cases is uncertain because disruption of ESCRT can also cause autophagy defects that have similar symptoms to autoimmunity, such as stunted growth, ROS and SA accumulation, elevated *PR* gene transcription, and aberrant cell death (Sertsuvalkul et al. 2022). Additional work is necessary to determine whether these cases are examples of autoimmunity or just autophagy defects.

## 6 Conclusion and Future Directions

It should not come as a surprise that ESCRT serves important roles in plant immunity because it is critical for a number of processes that are already known to contribute to pathogen resistance, most notably endosomal trafficking, MVB formation, and autophagy. Although these processes are known to rely on ESCRT, relatively few studies have specifically examined its role in regulating them in the context of immunity. One intriguing question worth exploring is whether ESCRT just rescues ubiquitinated, membrane‐bound immune signalling proteins from degradation or whether it also helps direct them to the appropriate subcellular location. Another lingering mystery is why ESCRT regulates the signalling of some NLRs but not others and whether the reason is related to differences in NLR subcellular localization. ESCRT's role in the formation of MVBs and EVs targeted to the infection site also deserves further study. Crucially, because ESCRT participates in diverse biological processes, its disruption is likely to produce pleiotropic effects and even lethality. For example, it is possible that autophagy defects associated with ESCRT dysfunction may obfuscate immune deficiencies caused by disruption of other ESCRT‐regulated processes unrelated to autophagy. To circumvent such obstacles, future studies may explore inducible systems to achieve conditional and/or spatiotemporal knockdown in combination with live cell imaging to better investigate ESCRT's roles in plant immunity.

ESCRT's importance in plant immunity is also evidenced by its targeting and manipulation by pathogens to create a favourable environment for pathogen growth and replication. The best‐characterized example of this is the exploitation of ESCRT by viruses to form VRCs. In animal systems, viruses also usurp the host cell ESCRT machinery for envelopment and cell exit, but whether plant viruses employ ESCRT for these processes remains unknown (Rivera‐Cuevas and Carruthers 2023). Besides viruses, intracellular bacteria and protozoan parasites have also been found to exploit host cell ESCRT in animal cells (Rivera‐Cuevas and Carruthers 2023). While it is unknown whether nonviral, intracellular plant pathogens also recruit host cell ESCRT, this seems likely given the prevalence of this strategy among intracellular pathogens. Multiple instances of nonviral plant pathogen effectors targeting plant ESCRT have also been documented. Considering ESCRT's involvement in multiple resistance mechanisms, it is likely that these effectors target ESCRT to suppress host immunity.

Further indication of ESCRT's importance in plant immunity comes from observations that its disruption can lead to autoimmunity, leading to the speculation that certain ESCRT components are ‘guarded’ by NLRs. However, interpreting these phenotypes is challenging, as ESCRT disruption can also result in impaired autophagy which has symptoms resembling autoimmunity, such as reduced stature and early senescence. Despite this difficulty, compelling evidence indicates that disruption of specific ESCRT components does indeed result in autoimmunity. In such cases, however, it is possible that ESCRT cargo is what is monitored by NLRs and not ESCRT itself. The discovery that the unviability of *amsh3* mutant plants is the result of severe autoimmunity and not developmental defects also raises the possibility that other ‘essential’ genes, including other ESCRT components, are actually guarded by the plant immune system. The seemingly paradoxical finding that autoimmunity can both be alleviated and induced by the loss of ESCRT components can be explained by the fact that not all NLRs require ESCRT for signalling.

In summary, a substantial body of evidence indicates that ESCRT complexes make positive contributions to plant immunity, while also functioning as susceptibility factors for certain pathogens such as viruses (Table 1). In the future, more work should be done to better understand ESCRT's various roles in plant immunity. Such knowledge would not only be helpful for developing pathogen‐resistant crops but may also yield information relevant for human health because ESCRT is highly conserved among eukaryotes.

## Author Contributions

**Willam Kyle Sexton:** conceptualization, writing – original draft, writing – review and editing. **Shunyuan Xiao:** conceptualization, writing – review and editing, supervision, funding acquisition.

## Funding

This work was supported by the National Science Foundation (IOS‐2224203).

## Conflicts of Interest

The authors declare no conflicts of interest.

## Data Availability

The data that support the findings of this study are available on request from the corresponding author. The data are not publicly available due to privacy or ethical restrictions.
